# Dr. Jackson, on the Virtues of Eye-Bright

**Published:** 1810-02

**Authors:** Robert Jackson


### *To the Editors of the Medical and Physical Journal.*

Gentlemen,

THE paper inserted in the Medical Journal of last May, on the virtues of cobweb, having, as it appears by some of your subsequent numbers, excited the wit and provoked the ridicule of some of your Correspondents, I am now, I believe, in the act of opening a new field for their raillery, if if you think proper to give the following communication to the public.

In the year 1800, when I was in Guernsey with the Russian auxiliary troops during their cantonment in that island, happening one day, in company with Dr. Walters of the British Hospital staff, to pass an old man on the road, who made an obeisance to the Doctor of more than usual respect, the Doctor mentioned to me casually, that the person, when nearly blind, had some time ago recovered his sight, by drinking a strong infusion of the herb eye-bright, which had been recommended to him by some of the neighbouring peasants. The fact was distinct, and the person who reported it was a man of the strictest veracity. I noted it down, but made no application of it till about three years since, when a woman at Stockton upon Tees, applied to me for advice on account of dimness of sight, rendering her almost unfit for her business, which was that of a house servant. She judged indistinctly of place and distance; and objects were, in fact, so obscure to her, that she scarcely discerned the difference between a knife and a fork as they lay upon a table. She was about thirty years of age; of a slender form, and, exclusively of the defect in vision, not in perfect health. The eyes were dim and filmy, but without pain or inflammation. I gave her a prescription, which was sent to the apothecary; and I further desired her to procure some eye-bright, and to drink about half a pint of the strong infusion morning and evening. I was not, I must confess, very sanguine in my expectations, that what I had ordered for her would do her much good; and I was therefore the more surprised to find, when I enquired for her about a month afterwards, that her sight was perfectly restored. The eyes were now bright and clear, and the general health was comparatively good.

I ascribed more to the eye-bright in the case which I have now stated, than to the other parts of the prescription; and the detail which I am now about to give of its effect on my own person, confirms me in my opinion. I am now on the verge of sixty, and have used spectacles for the last eight years. My sight was originally good; but it has declined so fast, that my spectacles are already of the fourth sight; and I could not, twelve months since, make out a paragraph in a common news-paper without their aid, scarcely even read a passage with the naked eye in books of the best type. I remembered what had been told to me by Dr. Walters, respecting the eye-bright, and thought I saw the report of its virtues verified in the case which has been noticed above; but I was desirous of further information on the subject, and for that purpose consulted the dispensatory. I there found that eye-bright had been recommended by Hildanus in strong terms, but that it was now generally, and according to the writer of the dispensatory, deservedly neglected. The dimness of my sight was a serious inconvenience, and its rapid progress in decay was rather alarming. The eye-bright presented itself to me daily last autumn, as I walked in the fields or lanes near my habitation; and I was thus, perhaps, urged by the frequent presentation to make an experiment which might decide, whether Hildanus and the vulgar, or Lewis and the faculty, were the best authority. With this view, I made a strong infusion from the dried leaves, and drank about half a pint of it at bed-time, and the same quantity early next morning. About one o'clock of the day, as my eye fell accidentally upon a news paper which lay on the table, near which I sat, I found it legible. I was struck with surprise; for, the day before, I could not have made out a single paragraph of it in any light in which it could have been placed. There was no visible cause to which the effect I have now noticed could be possibly assigned, except the eye-bright. It appeared to me to have begun an improvement, and I continued it three days longer, in expectation of further progress. At the end of three days, I could read the small army list, but not without difficulty. I then left it off, and at the end of a fortnight, my sight had so much declined, that I could scarcely make out a name in the army list, and I could not even read the news paper with satisfaction. I had recourse to it again, and continued it for six successive days; at the end of which, I was able to read the army list in a good light, and books of common type with facility in common light. I have repeated the experiment frequently, and always with similar effect.

My sight is renewed by at least three degrees, according to the measure of spectacles, since I commenced the use of the eye-bright; and though the clearness of vision declines after a time, it has not, even after the interval of a month, so far declined as to render spectacles absolutely necessary for common reading, though having been accustomed to read and write by their assistance, I still continue the habit. But besides the improvement, in so far as respects vision, the condition of the eyes is in other respects greatly more comfortable than it formerly was. Before I

had had recourse to the eye-bright, my eyes were frequently hot and painful, particularly in the evenings; the ball, when pressed, seemed flaccid and unequal; vision was often so obscure, that I was obliged to desist from reading, or seek relief in rubbing the eyes, or walking out into the open air; the sudden impression of light was painful, and accommodation to changes slow. At present, the eyes are rarely hot and painful in the evenings; the eye ball, when pressed, is comparatively equal, firm, and elastic; the impression of light striking suddenly is less unpleasant, and the eye more readily accommodates itself to changes than formerly. Under the immediate use of the infusion, and even for some time afterwards, all the objects in nature appear comparatively bright, as if they concentered an unusual portion of light. Vision is not so minute as it was at the early periods of life; but the clearness and fullness of objects, perhaps, as contrasted with the recent dimness and diminution, are singularly striking, and convey very pleasurable sensations.

To the case which I have now stated, and concerning results in which I am not likely to have been deceived, I shall add, that of a respectable farmer in the neighbourhood of Castle Eden, where I resided last autumn. This person had experienced, about twelve months ago, a kind of hemiplegic attack; that is, the muscles of the left side of the face, and of the left eye, were considerably affected; the mouth drawn to one side, the eye staring and distorted. There was besides general numbness in all the limbs; a want of power, but no actual paralysis; the memory was greatly impaired, and the powers of vision was so decayed, that he was incapable of reading a good type, even with spectacles of the fourth sight. He was a man of full habit, a muscular form, and naturally of a social disposition. The pulse was tense and strong; bark, wine, and tonics had been ordered for him, and continued for a length of time without benefit; I changed the plan of treatment entirely, and with evident good effect, on the general aspect of the disease. But as vision was particularly defective, perhaps more impaired than any of the other functions, I thought the case a fair one for the eye-bright, the benefits of which I then experienced in my own person. The herb abounded in the fields surrounding his house; and as it was known to him, I desired him to make a strong infusion from the dried leaves, and drink half a pint of it morning and evening. He did so; I saw him a few days thereafter. He was better in all

 respects; respects; but his eye-sight was astonishingly improved. It was nearly as good as it had been before his hemiplegic attack; that is, he could read easily by the help of his spectacles, and every thing around him appeared in his apprehension bright, distinct, and full. He left off the use of the infusion, and after a while, the distinctness and clearness of sight declined; he had again recourse to it, and experienced a similar improvement as at first.

From these facts, I leave it to the public to judge, whether the name of eye-bright has been given to this herb with reason, and whether or not, it is justly discarded from the catalogue of the regular practitioner. I mentioned its virtues to several persons; some tried it, and found benefit from it; others tried it without experiencing any sensible effect. It is perfectly innocent; it is not disagreeable to the taste; it is common in uncultivated fields and lanes, and it so directly renews the eye-sight, and so effectually and so agreeably ameliorates the condition of the stomach, that I cannot refrain from communicating this knowledge of its properties to the Editors of the Medical Journal, in the hopes that some poor man may thereby receive the benefit from it, which I have myself received.

I am, &c.

ROBERT JACKSON, M. D.

*January 1, 1810.*

